# Donor demographics and factors affecting corneal utilisation in Eye Bank of North India

**DOI:** 10.1007/s10792-021-01736-x

**Published:** 2021-04-24

**Authors:** S. K. Arya, Amit Raj, Jyoti Deswal, Piyush Kohli, Raghavendra Rai

**Affiliations:** 1 Department of Ophthalmology, Government Medical College and Hospital, Chandigarh, India; 2 Department of Ophthalmology AIIMS, Patna, Bihar India; 3 Regional Institute of Ophthalmology, PGIMS, Rohak, Haryana India; 4 Aravind Eye Hospital, Madurai, Tamil Nadu India; 5 Government Medical College and Hospital, Chandigarh, India

**Keywords:** Corneal blindness, Eye donor demographics, Eye donation, Eye bank, Hospital cornea recovery programme (HCRP)*

## Abstract

**Introduction:**

Nearly 6.8 million people in India have vision less than 6/60 in at least one eye due to corneal diseases; of these, about a million had bilateral involvement.

**Purpose:**

To identify the challenges faced; the trends in collection, storage and utilisation of corneal tissues in an eye bank in north India.

**Materials and methods:**

The past records of Eye Bank linked to a tertiary hospital in northern India were analysed from November’1999 to October’2015 with respect to number of eye donations per year, donor demographics and utilisation of corneal tissues.

**Results:**

The number of donations during the first 6 years were 100, 279 in the next 5 years and 473 in the last 5 years. The mean donor age was 63.2 ± 19.5 years. The percentage of donors less than 30, 31–60 and more than 60 years was 10%, 28% and 62%. Forty-two percent donations were from the hospital. The average time between the death and enucleation was 4.74 ± 5.31 hours. The percentage of corneas used in the donor age groups less than 30, 31–60 and above 60 years was 61.9%, 61.6% and 53.8%, respectively. The usability rate of the corneas from home and hospital was 63.7% and 55.3%, respectively.

**Conclusions:**

The eye bank had a lukewarm response in the beginning, but gained momentum with time. The myths and beliefs prevalent in our society deter people from donating eyes freely. Each eye bank needs to individualise its problems and find solutions for adequate procurement and utilisation of tissue.

## Introduction

World Health Organisation (WHO) had listed corneal opacities as the fourth leading cause of blindness accounting for 5.1% blindness globally [1]. Corneal blindness is estimated to be the second-most prevalent cause of blindness in developing countries and accounts for 98% of bilateral corneal blindness of the world [2]. Dandona et al.estimated that in 2001 there were approximately 6.8 million people in India with vision less than 6/60 in at least one eye due to corneal diseases; of these, about a million had bilateral involvement [3]. According to National Programme for Control of Blindness (NPCB) estimates, 25,000–30,000 corneal blindness cases are added in the country every year [4]. Even if the social practice of donating eyes rises to 1% of the death rate, the corneal transplant technology can wipe out corneal blindness from India [5].

The first eye bank in India was established at Madras by Dr Muthiah, who also performed the first corneal transplantation in India in 1948 [6]. India has made impressive progress since then. Over 700 eye banks, eye training centres and eye donation centres are currently registered in India [5]. But still, the number of corneal transplants done is far less than the actual requirement. Out of 700 eye banks registered, only 58 collect more than 100 corneas per year [5]. A number of programmes are being initiated to facilitate the expansion of eye banking. Two such programmes include the employment of professional eye bank managers and Hospital Cornea Recovery Programme (HCRP), which have stationed trained eye donation counsellors in large hospitals to approach potential donor families to gain consent [2].

This study was done to identify the challenges faced; the trends in collection, storage and utilisation of corneal tissues in an eye bank in north India.

## Material & methods

**Fig. 1:**
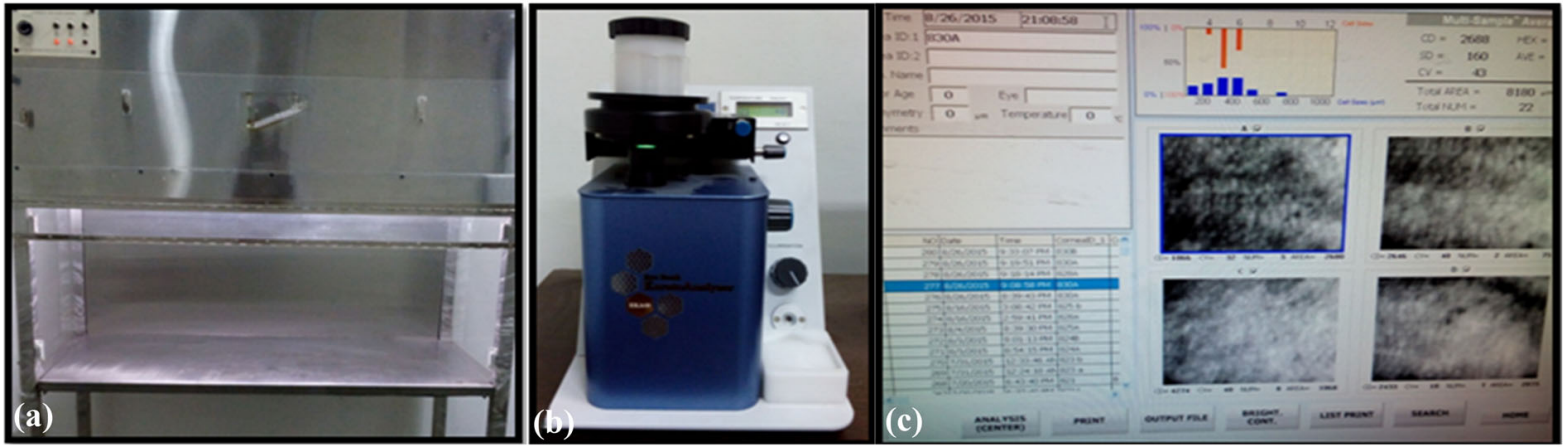
Clinical photographs showing the **a** Laminar flow used to remove corneas from the eye balls under aseptic conditions; **b** Eye bank analyser EKA-10; and **c** Software used KSS-EB10 Version 3.01 used to count endothelial count

This retrospective study was conducted at a tertiary hospital in northern India. The study adhered to the tenets of the Declaration of Helsinki. As per the policy of the institution, retrospective studies receive waiver for ethical clearance. An informed consent was taken from at least two authorised representatives of the donors. The records of the associated Eye Bank were analysed from November 1999 to October 2015 with respect to the number of eye donations per year, donor age, sex, cause of death, rural or urban background of the patient, source of donation, place of eye donation, time gap between time of death and enucleation and utilisation of corneal tissues. The corneas were graded on the slit lamp according to Orbis telemedicine guidelines The corneal endothelium was then evaluated with the help of specular microscopy [7]. Corneas with endothelial count less than 1500cells/mm^2^, severe polymegathism or pleomorphism, presence of central corneal guttata were not used for ocular penetrating keratoiplasty (OPK) [8]. Konan eye bank specular microscope KeratoAnalyzer EKA-10 with software KSS-EB10 (Konan Medical USA, Irvine, CA) is currently being used (Fig. 1). The corneas were discarded in case of poor-quality or medical contraindications for usage of cornea as given by NPCB (Standards of Eye Banking in India, 2009).

### Statistical analysis

The data was entered into a Microsoft Excel spreadsheet and statistical analysis was performed with STATA statistical software, Version 14.0 (StataCorp, College Station, Texas, USA). Means and standard deviations for continuous variables and proportions for categorical variables were calculated. Proportions were compared using Chi-square test, while means were compared using t test and ANOVA.

## Results

### Donation over the years

**Fig. 2:**
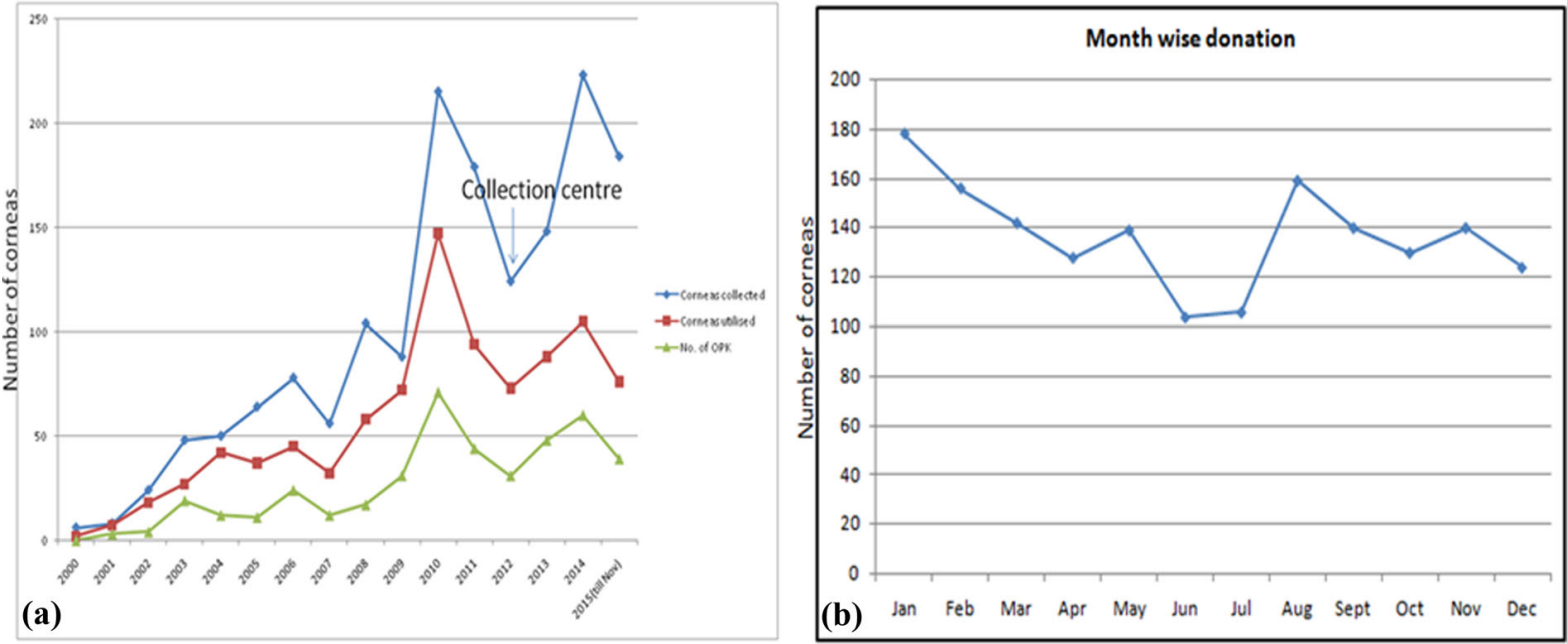
Graphs highlighting the **a** year-wise collection and utilisation of corneas; and **b** month-wise collection of corneas

A total of 1646 corneas were received from 851 donors as 56 single eyeballs were received from other eye banks and collection centre. Three hundred and fifty-five donors (41.7%) were females while 496 (58.3%) were males. The number of corneas received during the first 6 years was 200 with an average of 33.3 per year, 541 corneas in the next 5 years at an average 108.2 per year while the last 5 years yielded 905 corneas at an average of 181.0 per year (Fig. 2a). Since the start of one collection centre 3 years ago, the average donations have risen to 200.7 per year (total 602). The collection centre contributed at an average of 73.3 corneas per year (total 220). The minimum number of donations each year were always during June to August contributing 369 (22.4%) donations and showed an increasing trend in the next two quarters with September to November contributing 416 (25.3%) donors and December to February accounting for 452 (27.5%) donors, while March to May showed a slight dip with 409 (24.8%) donors. (Fig. 2b).

### Age profile of the donors

**Fig. 3:**
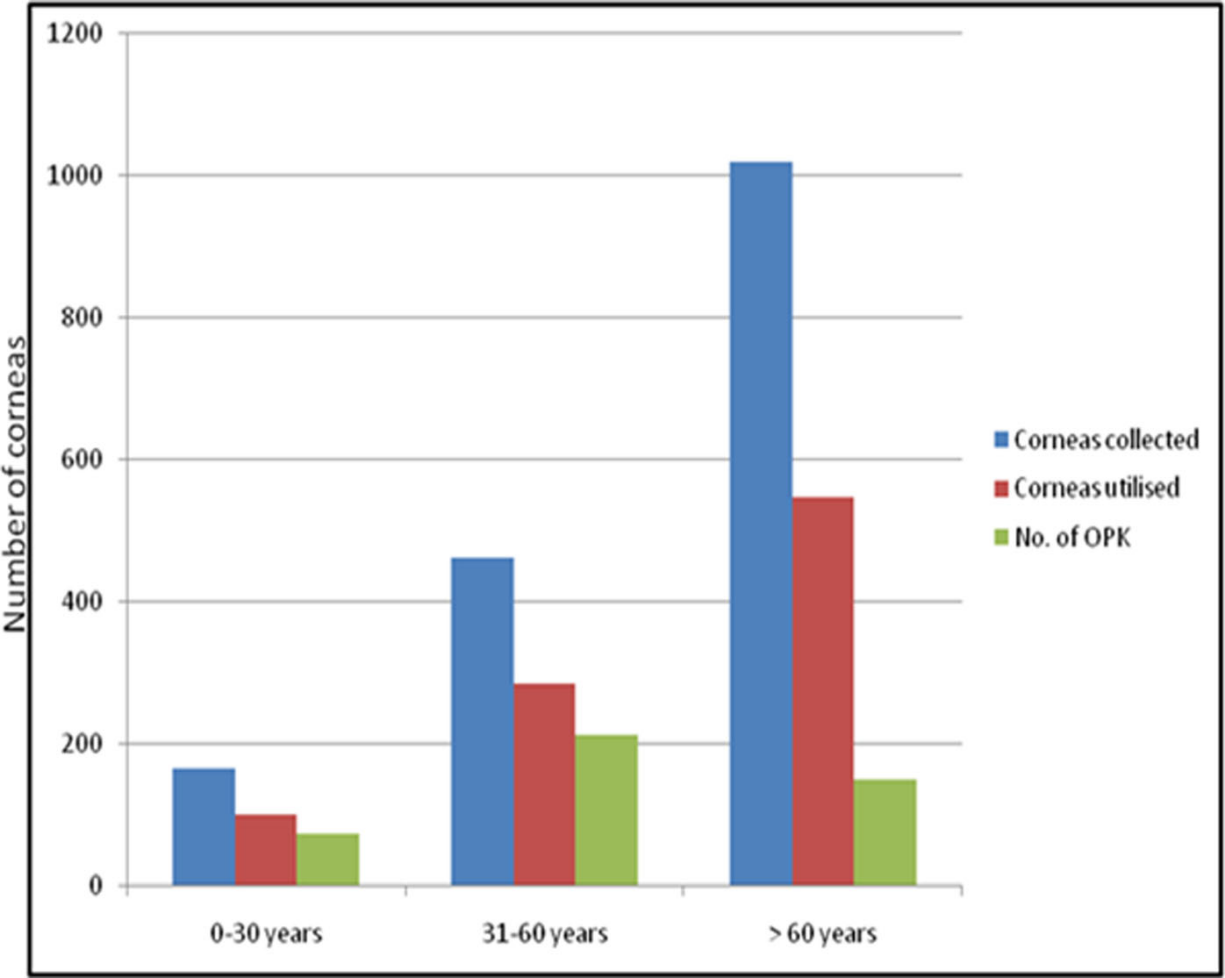
Graphs highlighting donor age-wise collection and utilisation of corneas

The mean donor age was 63.2 ± 19.5 years while for males it was 61.0 ± 20.0 years and for females it was 66.0 ± 18.5 years. While the minimum donor age was 49 days, who died of septicaemia; maximum age was 102 years. The mean donor age increased with passing years of eye banking. It was 54 ± 18.9, 63 ± 18.8 and 65 ± 19.5 years, respectively, in the first 6, middle 5 and the last 5 years of eye banking. Sixty-two percent (*n* = 529) donors were above the age of 60 years, 28.0% (*n* = 238) between 31 and 60 years and 9.9% (*n* = 84) below 30 years of age (Fig. 3). There were only 3 donors with age less than 10 years. The maximum number of donors were in age group of 71–80 years (*n* = 205, 24.0%) followed by 61–70 years (*n* = 175, 20.5%).

### Cause of death

While 27.8% patients (*n* = 237) died a natural death because of old age, 13.2% (*n* = 112) died an unnatural death due to road side accidents, poisoning, burns, hanging, murder, etc. and 59.0% (*n* = 502) died of some medical illness. Three leading causes of deaths due to medical illness were cardiovascular diseases, septicaemia and liver cirrhosis.

### Time of donation

Majority of donations (62%, *n* = 532) were taken during day hours i.e. 8am to 8 pm while 38.0% were during the night duty hours i.e. 8 pm to 8am. On the contrary, 63.9% (204/319) donations during the night duty hours were from outside the hospital, it was 54.3% (289/532) during the day hours. The attendants of the patients dying in hospitals are less receptive towards the idea of eye donation during night hours, while the pledged donors do not have such a bias.

### Rural urban profile of donors

**Fig. 4:**
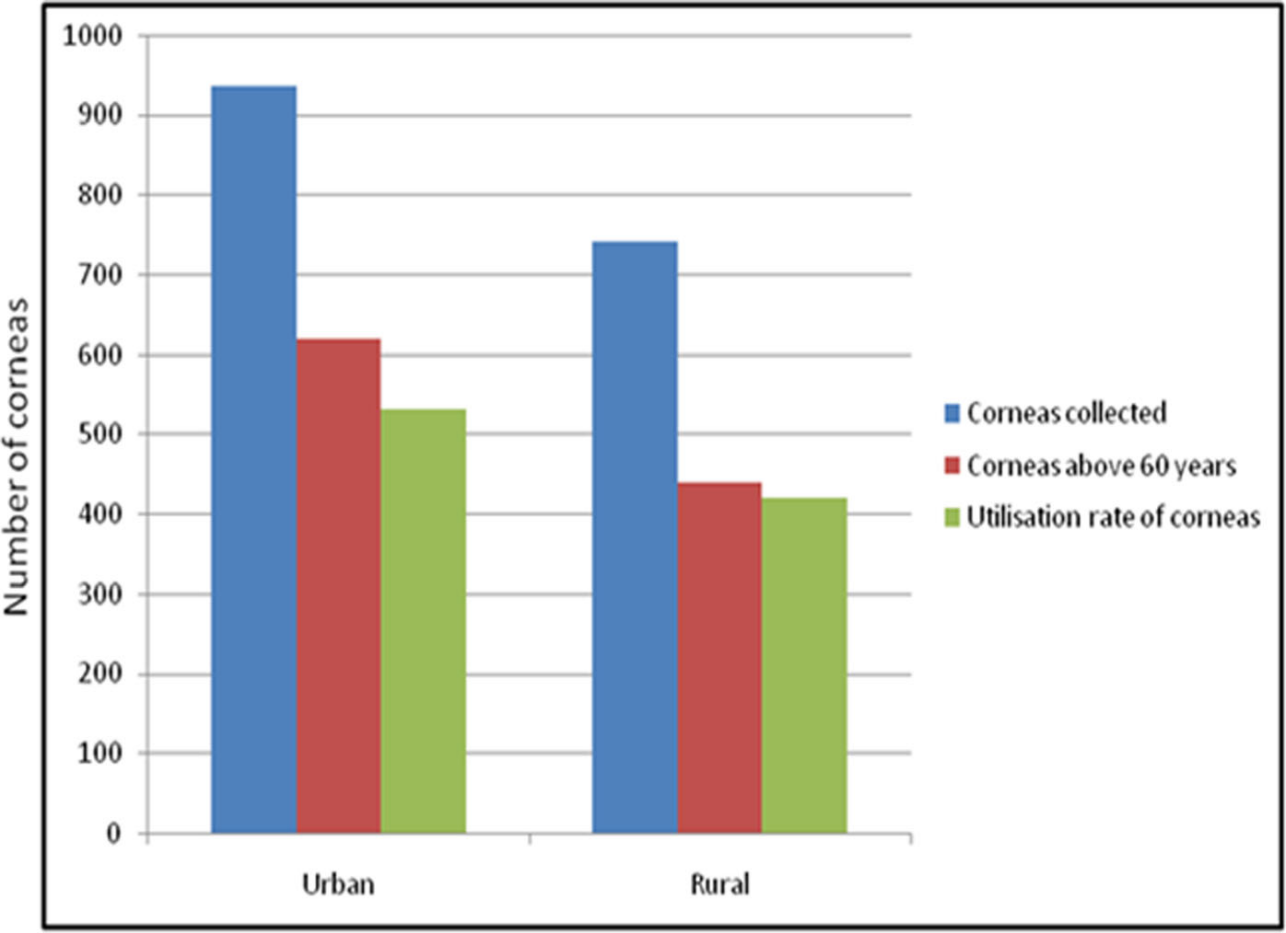
Graphs highlighting donor background-wise collection and utilisation of corneas

While 489 donors (57.5%) were from urban background, 362 (42.5%) were from rural background. The average donor age from urban and rural background was 64.0 ± 19.6 years and 62.5 ± 19.2 years respectively, which was again a statistically significant difference (Fig. 4). Proportion of urban donors in age groups of less than 30 years, 31–60 years and more than 60 years was 56.8%, 55.5% and 58.4%, respectively. Out of 3 donors below the age of 10 years, two were from urban background and died due to road side accident, while one was from rural background and died due to septicaemia.

### Source of donation

**Fig. 5:**
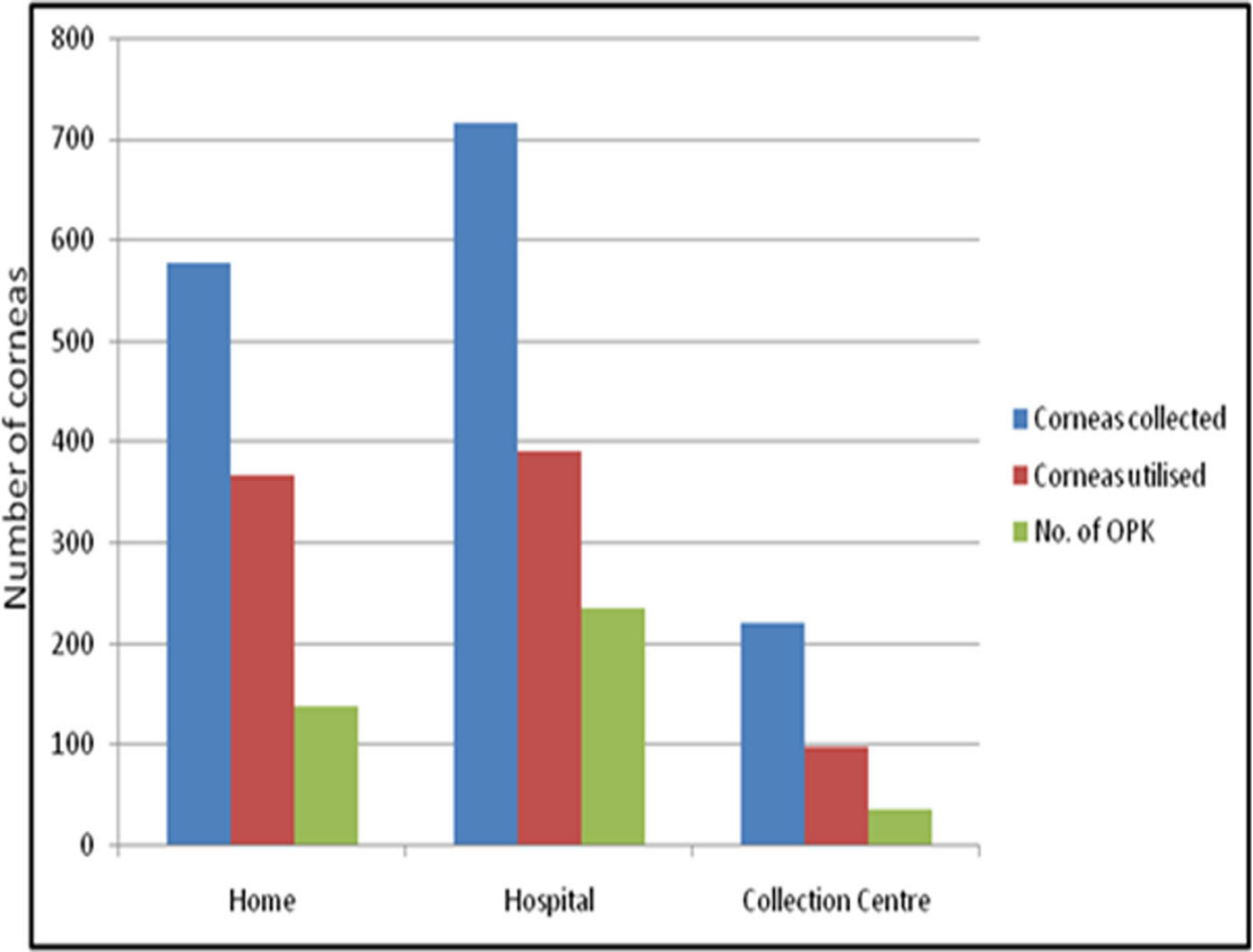
Graphs highlighting donor source-wise donations and utilisation of corneas

**Fig. 6:**
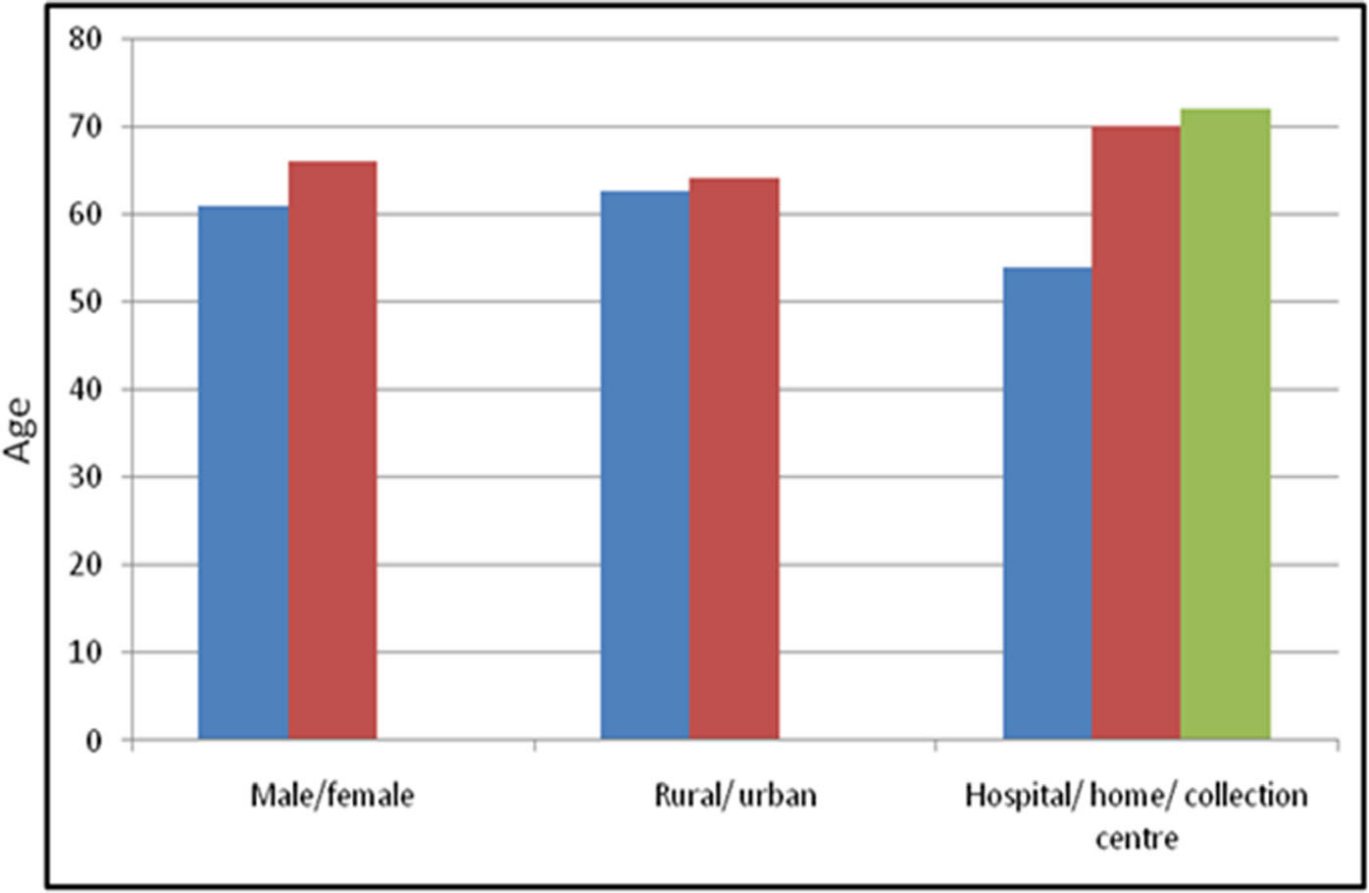
Graphs comparing the average age of donor as per gender, background and source

Thirty-nine percent (335/851) of donations were collected from home (including old age home), 42% (*n* = 358/851) from the hospital itself, 12.9% (*n* = 110/851) from the collection centre, 5.6% (*n* = 48/851) from other eye banks (Fig. 5). The average donor age from hospital, home and collection centre were 54.0 ± 20.8, 70.0 ± 14.2, 72.0 ± 16.3 years respectively (Fig. 6). The donors less than 60 years were mainly from the hospital. Proportion of donations in age groups less than 30 years and 31–60 years that came from the hospital were 79.8% (67/84) and 59.7% (142/238). Among donors above the age of 60 years, 45.0% were from home while 28.2% were from the hospital. The donations received from collection centre and other eye banks had highest proportion of donors from age groups of more than 60 years 83.6% (92/110) and 70.8% (34/48), respectively.

### Average time between the death and enucleation

Average time between the death and enucleation was 4.74 ± 5.31 h. Among the donations from the hospital, it was 5.39 ± 7.37 h while that from outside it was 4.26 ± 2.97 h and the difference was statistically significant. This was mainly because of two reasons. Firstly, 25.7% (92/358) donations from the hospital were Medico-legal cases (MLC) and required clearance from the police before enucleation could be carried out. While the time gap between in MLCs was 12.6 ± 7.38 h; in other cases it was 3.28 ± 5.90 h. Secondly, the guardians usually keep the dead bodies in the mortuary while arranging for transportation. This gives the counsellors an opportunity for interaction, but the donation is usually delayed. Average time during day hours was 4.45 ± 5.99 h while during night hours it was 5.21 ± 3.89 h, which is a statistically significant difference.

### Utilisation of corneas

Seven hundred and thirteen (43.3%) corneas were discarded either due to poor-quality or medical contraindication due to the cause of death; while 933 corneas (56.7%) were used out of which 436 (26.5%) were used for OPK and rest for Therapeutic Penetrating Keratoplasty (TPK). In the first 6 years, usability rate was 66.5% (133/ 200); in the next 5 years, it was 65.4% (354/541) while in the last 5 years it reduced to 46.9% (406/865). This was mainly because with passing years more old-aged and poor-quality corneal donations increased at a higher rate. Out of 713 corneas discarded, 458 (64.2%) were discarded during the last 5 years. On the contrary, percentage of corneas used for OPK was 24.5%, 17.9% and 26.8% in the first 6 years, next 5 years and the last 5 years respectively (Fig. 2a).

### Donor age-wise utilisation of cornea

**Fig. 7:**
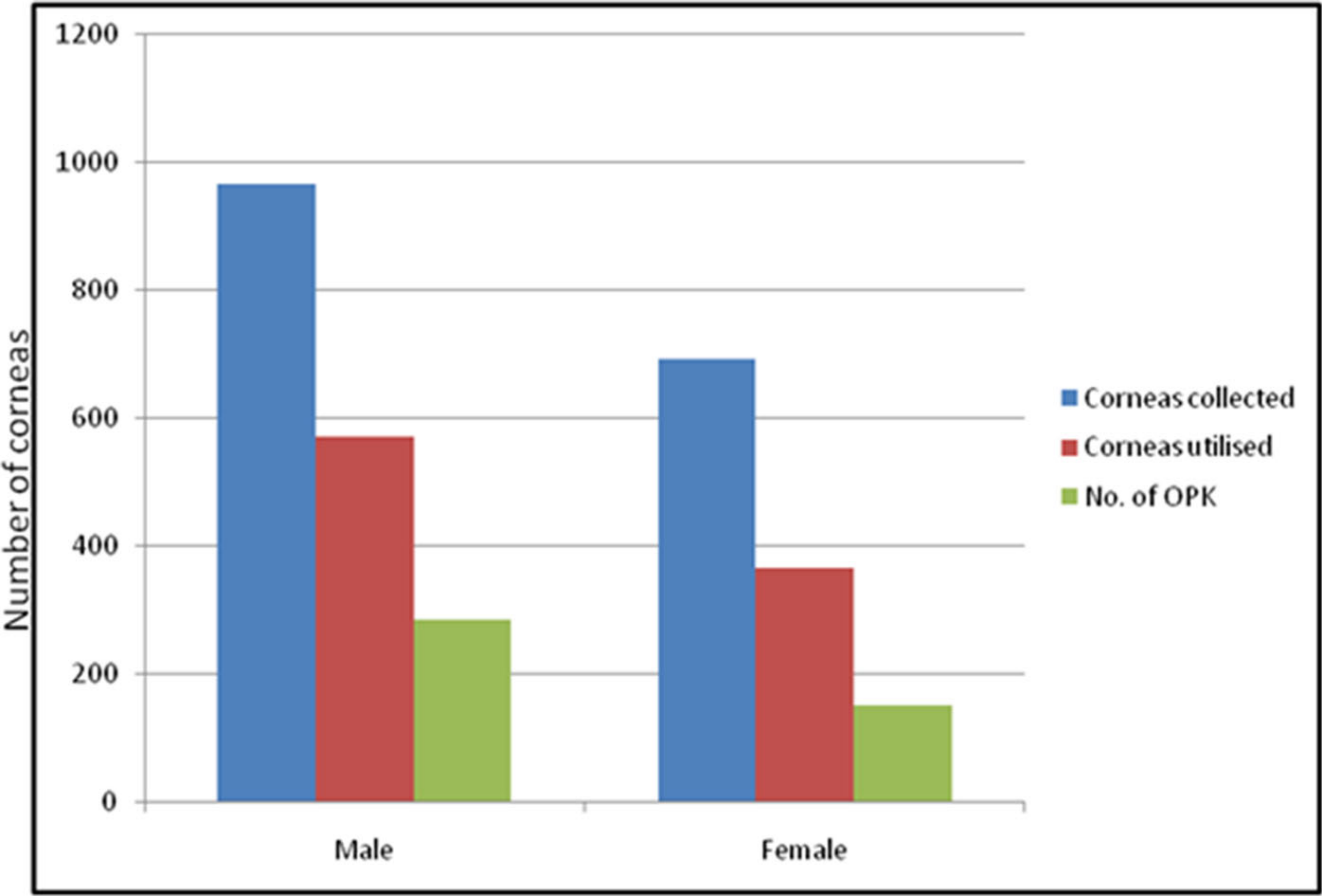
Graphs highlighting donor gender-wise donations and utilisation of corneas

Out of the six corneas of donors less than 10 years, four were discarded while two were used. With increase in the donor age, percentage of usability of corneas decreased. The percentage of corneas used in the donor age group less than 30, 31–60 and above 60 years was 61.9%, 61.6% and 53.8% respectively. Percentage of corneas used for OPK in the donor age groups less than 30, 31–60 and above 60 years was 45.6%, 46.0% and 14.8% respectively, as the quality of donor cornea declined with the increasing donor age (Fig. 3). The percentage of corneas discarded due to medical contraindication in donor age group less than 30, 31–60 and above 60 years was 30.0%, 21.9% and 14.6% respectively. This was because majority of young people had an unnatural death. A higher percentage of the male corneas were used (59.2%) as compared to the female (53.1%) ones (Fig. 7). This was because the average age of female donors was higher.

#### Source-wise utilisation of cornea

The usability rate of corneas from urban (56.9%) and rural (56.6%) donor corneas was similar. The percentage of corneas used from those collected from home, hospital, other eye bank and collection centre was 63.7%, 55.3%, 55.7% and 44.3% respectively. Hence, highest yield of usable corneas was from home while the lowest yield was from the collection centre. This was because the average donor age from collection centre was far higher than the average donor age while the corneas from hospital had to be discarded due to medical contraindication. The percentage of corneas used for OPK from those collected from home, hospital, other eye banks and collection centre was 23.8%, 32.8%, 13.9% and 16.0%, respectively (Fig. 5). Hence, the highest yield of corneas used for OPK was from hospital while the lowest yield was from the collection centre. This was because average of donors from hospital was the least (54.0 ± 20.8 years) while it was highest for the collection centre (72.0 ± 16.3 years).

## Discussion

This eye bank had a lukewarm response in the beginning, with only 14 corneas in the first 2 years. But with time it gained momentum. The addition of a collection centre has had a significant impact on the corneal collection. Corneal collection all over India showed a 2.5-fold increase from 20,451 to more than 50,000 from 2003 to 2013. [5] Our eye bank had a better performance and showed a fourfold increase from 48 to 198 corneas over the same period. There was an increase by 42.0% of corneal collection by north zone from 2007 to 2008 as they increased from 3675 to 5208 while our eye bank showed an increase of 87.0% in cornea collection increasing from 112 to 208 [9]. One of main reasons behind this regular increase has been the introduction of HCRP. The “grief counsellors” have been able to successfully motivate the family members of the deceased individuals to donate cornea. The programme has helped to increase the availability of deceased donors’ medical history, reducing the time interval between death and tissue harvestation, and increase the availability of young donors [10].

Apart from the daily efforts, “Eye donation fortnight” is celebrated every year from 25th August to 8th September, during which a lot of advertisement and awareness programmes are conducted to sensitise people for eye donations, especially targeting schools and colleges. The number of donations showed an increasing trend for the next 6 months after this fortnight, before dipping again.

The donor age distribution was comparable to the published results from other eye banks with the majority of donors being over 60 years of age (Fig. 3), and the greatest proportion between 70 to 80 years of age [11, 12]. Majority of the increase in donation has been from the old-aged ones. It is often difficult to convince parents who are in a state of emotional shock after the death of their young children and have low receptivity to the idea of eye donation. It must be understood that time of soliciting is not an opportunity for motivational effort. The results of our study suggest that it would be better to spread awareness at school and college level, while making the young people pledge their eyes after their death, especially if untimely. During the time of grief counselling and moral support, the counsellors should tactfully remind the parents about eye donation. Also, hospital administration needs to promote a policy that calls for requesting donation permission from all who die within the hospital unit.

The male dominance (Fig. 7) is also similar to the results earlier published [12]. The interval between death and tissue harvestation is much higher in case of MLCs . This delay is similar to that reported in other Indian studies [13]. As most of the MLC cases yield young corneas, effort needs to be made to reduce this time by hastening the process of formality completion.

The utilisation rate of corneas (Fig. 1a) for our eye bank in the year 2013 was 49.5% (98/198), while it was around 46.0% for the whole country with around 23,000 keratoplasties done from around 50,000 corneas collected [5, 13]. Eye bank Association of (EBAI) India has set a target of 1 lac transplants annually to be achieved by 2020 [5]. With an annual utilisation rate of approximately 50%, 2 lac corneas are needed. Based on 10.2 million deaths in 2013–14, we need corneal donation from only 1% of total deaths [5]. In our eye bank, 44.5% (358/803) donations were obtained through HCRP while rest were voluntary donations. According to the EBAI, overall Indian eye bank utilisation of tissue through primarily voluntary collection is 38% while utilisation within a HCRP model is 72% [2]. On the contrary, our eye bank had about 55% utilisation in both scenarios. The main reasons for low utilisation rates were poor-quality corneas due to old age of the donors and unavailability of appropriate recepient  at the time of donation. Although the quality of old-aged donors is often poor, we cannot refuse donation as this can lead to the spread of rumours and myths, further impacting the trend of donations. The non-viable corneas were used for research and training purposes. We tried to reduce tissue wastage by distributing the available surplus tissue to other eye banks and vice versa. The opening of collection centre is one such example of the link programme. However, in unavoidable circumstances, the precious tissues sometimes did go underutilised owing to logistical difficulties.

Though the corneal donation has increased, we still need alternate plans to increase the quantity and improve the quality of the tissue collected. Myths and beliefs prevalent in our society deter people from donating eyes freely [14, 15]. On the contrary, Sri Lanka by propagating the manuscript mentioning the instance of eye donation in Buddhist scriptures has made eye donation not only culturally acceptable but even desirable. Similarly, Nepal has shifted the tissue procurement centre to the Pashupati Temple, located on the banks of Bagmati River, where the deceased in Kathmandu are cremated. As a result, there has been a five-fold increase in donations as well as quality of donated cornea has improved [16]. We should follow similar path as taken by our neighbours and collaborate with local social, religious and spiritual leaders to publicly provide culturally specific rationales and promote the practice of eye donation.

Gain et al. *in* their survey highlighted the influence of religion on corneal donation [17]. Priyadarshini et al. highlighted that illiteracy and rural residence were strong predictors of ignorance about eye donation [18]. However, Tandon et al. reported that literacy and socioeconomic scale did not influence the willingness for eye donation. As per their report, the major reasons for refusing donation were dissuasion by distant relatives, legal problems and religious beliefs [14]. More Indian studies need to be conducted to look for multicultural aspects that influence donation. This will help us understand the myths and beliefs prevalent among the people according to their ethnical differences like religion, caste, education.

We share our 16-year experience with eye banking. Inclusion of collection centres, local propaganda with help of Non-government organisations (NGOs), spreading awareness at the level of schools and colleges, and exchange of corneas with nearby eye banks in case of non-availability of recepients has increased the utilisation of tissue. Apart from the central programmes, each eye bank needs to individualise its problems, find solutions and plan for increasing the quantity, quality and utilisation of the tissue.

## References

[1] World Health Organisation (2015) Priority of eye diseases. [ONLINE] http://www.who.int/blindness/causes/priority/en/index8.html..

[2] Oliva M, Gulati M, Schottman T (2012). Turning the tide of corneal blindness. Indian J Ophthalmol.

[3] Dandona P, Qutob T, Hamouda W, Bakri F, Aljada A, Kumbkarni Y (1999). Heparin inhibits reactive oxygen species generation by polymorphonuclear and mononuclear leucocytes. Thromb Res.

[4] National Programme for Control of Blindness [Internet]. Standards of Eye Banking of India 2009 [Cited 2016 Feb 25]. http://npcb.nic.in/writereaddata/mainlinkfile/file176.pdf

[5] Basak S K. Updating from Eye Bank Association of India. [ONLINE] http://www.gaeba.org/wp-content/uploads/2014/01/Session-1-Basak.pdf

[6] Kannan KA (1999). Eye donation movement in India. J Indian Med Assoc.

[7] Mathur V, Parihar JKS, Srivastava VK, Avasthi A (2013). Clinical evaluation of Deep Anterior Lamellar Keratoplasty (DALK) for stromal corneal opacities. Med J Armed Forces India.

[8] Saini JS, Reddy MK, Sharma S, Wagh S (1996). Donor corneal tissue evaluation. Indian J Ophthalmol.

[9] Eye Bank Association of India [Internet]. Punarjyoti newsletter September,04-April,08; Volume IX, No 1 [Cited 2016 Feb 25]. http://ebai.org/images/resources/Punarjyoti_News_Letter.pdf

[10] Chaurasia S, Mohamed A, Garg P (2020). Thirty years of eye bank experience at a single centre in India. Int Ophthalmol.

[11] Eye Bank Association of America [Internet]. 2012 Eye Banking Statistical Report. Washington DC [Cited 2016 Feb 25]. http://www.restoresight.org/wp-content/uploads/2013/04/2012_Statistical_Report_FINAL-reduced-size-4-10.pdf

[12] Patel HY, Brookes NH, Moffatt L (2005). The New Zealand National Eye Bank study 1991–2003: a review of the source and management of corneal tissue. Cornea.

[13] Sharma N, Arora T, Singhal D (2019). Procurement, storage and utilization trends of eye banks in India. Indian J Ophthalmol.

[14] Tandon R, Verma K, Vanathi M (2004). Factors affecting eye donation from postmortem cases in a tertiary care hospital. Cornea.

[15] .Gupta N, Vashist P, Ganger A, (2018). Eye donation and eye banking in India. Natl Med J India.

[16] Ruit S, Tabin G, Gurung R, Shattuck T, Murchison A, Dimmig J (2002). Temple eye banking in Nepal. Cornea.

[17] Gain P, Jullienne R, He Z (2016). Global survey of corneal transplantation and eye banking. JAMA Ophthalmol.

[18] Priyadarshini B, Srinivasan M, Padmavathi A (2003). Awareness of eye donation in an adult population of southern India. A pilot study Indian J Ophthalmol.

